# Halogen Bonds under Electric Field with Quantum Accuracy and Relativistic Basis Sets

**DOI:** 10.1021/acs.jpca.5c08038

**Published:** 2026-01-04

**Authors:** Gabriele Ottanà, Simona Mastronardo, Petr Eminger, Klaudia Mráziková, Sebastiano Trusso, Franz Saija, Martin Ferus, Luigi Monsù Scolaro, Jing Xie, Matteo Tommasini, Giuseppe Cassone

**Affiliations:** † Department of Chemical, Biological, Pharmaceutical, and Environmental Sciences, 18980 University of Messina, 31 Viale F. Stagno d’Alcontres, 98166 Messina, Italy; ‡ Heyrovský Institute of Physical Chemistry, Academy of Sciences of the Czech Republic, Dolejškova 3, CZ18223 Prague 8, Czech Republic; § Faculty of Science, Department of Physical and Macromolecular Chemistry, Charles University in Prague, 12840 Prague, Czech Republic; ∥ Institute for Chemical-Physical Processes, 9327 National Research Council of Italy (CNR-IPCF), V. Stagno d’Alcontres 37, 98158 Messina, Italy; ⊥ Ministry of Education Key Laboratory of Cluster Science, Beijing Key Laboratory of Photoelectronic/Electrophotonic Conversion Materials, School of Chemistry and Chemical Engineering, 47833 Beijing Institute of Technology, 100081 Beijing, P. R. China; # Dipartimento di Chimica, Materiali e Ing. Chimica “G. Natta”, 18981 Politecnico di Milano, Piazza Leonardo da Vinci, 32, 20133 Milano, Italy

## Abstract

Halogen bonds (XBs) are a cornerstone of supramolecular chemistry, yet their response to external perturbations remains poorly investigated, particularly in systems with heavy elements where relativistic effects are significant. We benchmark two prototypical iodine-chloride X-bonded complexes, ClI···N­(CH_3_)_3_ and ClI···NCH, under electric fields (EFs) using quantum chemical calculations up to CCSD and CCSD­(T). Relativistic basis sets, including the all-electron jorge-TZP-DKH, are assessed against non-relativistic and pseudopotential-based alternatives (def2-TZVP, SDD, LANL2DZ) for their impact on XB geometries, binding energies, vibrational Stark shifts, and electron density redistribution. Explicit relativistic treatments substantially reduce the exaggerated field response otherwise observed. Benchmarking M06-2X and B3LYP with various basis sets against correlated methods confirms the accuracy of M06-2X, while showing that the relativistic effects included in the basis set influence the results more than the choice of functional itself. Symmetry-Adapted Perturbation Theory (SAPT) indicates that electrostatics dominate XB stabilization, with induction becoming more relevant under strong positive fields. Overall, XBs prove markedly more sensitive to external EFs than H-bonds across different field arrangements.

## Graphical abstract

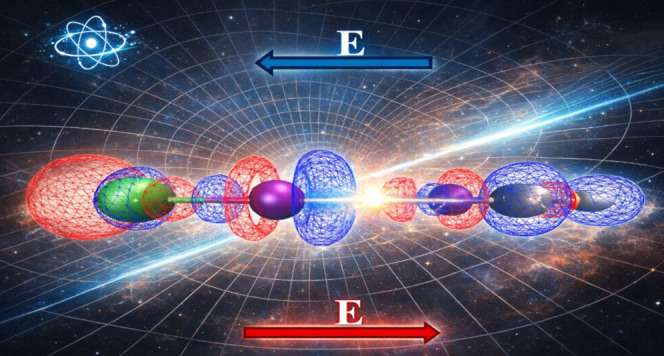

## Introduction

Halogen atoms (denoted in this paper as X) often act as electrophilic centers in noncovalent interactions. Once covalently bound, a region of lower electron density with a typically positive electrostatic potential, called the σ-hole, occurs opposite to the covalent bond and is surrounded by a belt of higher electron density.^1,2^ This positive electrostatic region interacts with nucleophilic sites (e.g., lone pairs or anions) to form halogen bonds (XBs).

Although certainly less well-acknowledged than the hydrogen bond (H-bond), the concept of X-bonding can be traced back to early observations in 1814.^3^ Nevertheless, the formal terminology was introduced only in 1961.^4^ The IUPAC definition describes an X···Y halogen bond as an attractive interaction of the type R–X···Y, where X is the halogen, R is a covalently bonded substituent, and Y is an electron-rich site.^5^ These interactions are highly directional, often approaching linear geometries (∼180°), whereas their strength generally increases with the polarizability of the specific halogen atom involved. Recent experimental breakthroughs have provided unprecedented insight into the nature of X-bonding. In particular, the first real-space visualization of σ-holes was achieved using Kelvin probe force microscopy,^6^ confirming the presence of the electron-deficient region predicted by theory.

X-bonding has found widespread applications across crystal engineering, supramolecular chemistry, and drug design.^7–9^ Computational chemistry has been essential in these advances, elucidating how substituents tune XB stability,^10^ influencing molecular conformations,^11^ and guiding drug-development strategies.^12^ For a comprehensive overview of applications, the interested reader can refer to the review by Cavallo et al.^1^ The binding energy of XBs arises from a complex interplay between electrostatics, polarization, dispersion, and charge transfer contributions.^13^ Early models emphasized electrostatics and charge transfer, drawing analogies with H-bonding.^14–16^ However, more recent investigations underscore the crucial role of London dispersion.^17,18^ In particular, Riley and Hobza, by exploiting Symmetry-Adapted Perturbation Theory (SAPT),^19^ demonstrated that both dispersion and electrostatic effects crucially stabilize X-bonded complexes.^18,20^ Similarly, robust benchmark calculations by Kozuch and Martin^21^ and Siiskonen and Priimagi^22^ further emphasized that X-bonding is not purely electrostatic in nature, but instead a multicomponent interaction where dispersion often contributes substantially. This view has been reinforced by comprehensive SAPT analyses,^18^ which revealed the balance of different energy terms depending on the specific chemical environment.^10,18,20^

In recent years, the development of high-resolution techniques such as scanning tunneling microscopy (STM) and atomic force microscopy (AFM) has enabled the manipulation of intermolecular bonds at the molecular scale.^23–27^ The application of tailored external electric fields (EFs) has revealed unprecedented catalytic scenarios, demonstrating the ability of EFs to steer and accelerate a wide variety of chemical transformations.^28–32^ In parallel, computational investigations have provided fundamental insights into the interplay between external EFs and H-bonds in molecular and supramolecular systems.^33–43^ Although to a lesser extent, also XB systems exposed to EFs were investigated by means of Density Functional Theory (DFT) simulations. In fact, Shaik and co-workers demonstrated that oriented external EFs, when aligned along the XB axis, act like electric tweezers that significantly lower reaction barriers enabling nucleophilic displacement reactions otherwise inaccessible in both gas and solution phases.^44^ Furthermore, recent investigations show that the stability and geometry of C–X···π XBs are highly sensitive to both the strength and orientation of the applied field, with heavier halogens (Br, I) exhibiting the strongest effects.^45^ Energy decomposition analyses revealed that stabilizing fields enhance the electrostatic contribution by reinforcing σ-hole polarization, whereas opposite fields weaken or destabilize the interaction. In addition, a more recent computational study investigated how homogeneous external EFs influence intrinsic XB strengths, deconstructing the effects into steric and orbital interaction components.^46^

Interfacial EFs in microdroplets (∼0.1–1 V/Å) may also contribute to the activation of dihalogen molecules (X_2_) by nucleophiles (Nu), as a suitably oriented field can facilitate electron transfer from Nu to X_2_, thereby generating (NuX)^+^ and X^–^. The latter may subsequently react with an additional X_2_ to yield X_3_^–^, with the entire process being strongly facilitated by field alignment along the XB axis.^47^ However, other factors might be at play since recent investigations mitigate the role played by interfacial EFs in water microdroplets.^48–52^ Interestingly, high-resolution molecular beam scattering has been used to disentangle the fundamental contributions of hydrogen and halogen bonding, taking advantage of the electric fields emerging at large intermolecular separations.^53^

Notwithstanding the relevance of those computational works, to the best of our knowledge, no systematic benchmark up to the coupled cluster single and doubles (CCSD) and perturbative triples CCSD­(T) levels and exploiting relativistic basis sets, has been reported so far. This gap is noteworthy, since iodine comprises 53 electrons, a number far exceeding the empirical threshold of 36 beyond which relativistic effects are expected to become non-negligible.^54^ In the present work, we perform such a benchmark across different DFT functionals, up to the CCSD and CCSD­(T) levels of theory, using non-relativistic, pseudopotential-based relativistic, and all-electron relativistic basis sets on two X-bonded systems exposed to oriented EFs. Moreover, a detailed SAPT analysis as a function of field strength and direction is presented.

## Methods

To benchmark the accuracy of functionals belonging to different Perdew Jacob’s ladders^55^ we have investigated at different Density Functional Theory (DFT) levels the properties of two halogen-bonded (X-bonded) dimers formed by iodine monochloride ICl—namely, with trimethylamine [N­(CH_3_)_3_] and hydrogen cyanide (HCN)—subjected to static and homogeneous external electric fields (EFs). In particular, we selected for our comparison the hybrid B3LYP^56–59^ and the hybrid *meta*-GGA M06-2X^60^ exchange–correlation functionals. The DFT results were then compared with more accurate geometries and energies obtained from wave-function based methods. In particular, we have computed in the presence of external EFs of various strengths and directions the gas-phase optimized molecular geometries at singles and doubles coupled cluster method (CCSD),^61–64^ whereas we used the perturbative triples method CCSD­(T)^65–67^ for the energies on CCSD geometries.

As anticipated, we also carried out a benchmark over different basis sets to assess the impact of relativistic effects. On the one hand, we used the non-relativistic Pople’s 6-311++G­(d,p) and the Dunning correlation-consistent *aug-cc-pVTZ* ^68^ basis sets. On the other hand, we used the semiempirical *LANL2DZ*,^69^ the *SDD*,^70^ and the Weigend & Ahlrichs *def2-TZVP*,^71,72^ including small-core effective core potentials which embed scalar relativistic effects into the pseudopotentials, and we compared them with the purely all-electron relativistic basis set *jorge-TZP-DKH* that was obtained through the use of the Douglas–Kroll–Hess relativistic Hamiltonian.^73,74^

For each combination of method and basis set, geometry optimizations were performed for both XB dimers under investigation. The initial calculations were performed in the absence of the external EF, whereas the remaining computations were executed in the presence of a field oriented parallel to the global dipole moment of the two molecular systems here considered (Figure 1). The applied EF is here considered as “positive” when it leads to an increase of the overall dipole moment of the system and, viceversa, as “negative” when it leads to a decrease of it. We explored the impact of external EFs on both systems by considering field strengths symmetrically in the range from −1.00 to +1.00 V/Å. Around the zero-field value, relatively small field increments of ±0.05 V/Å and ±0.10 V/Å were used, whereas larger steps of ±0.25 V/Å were employed farther from the zero-field region. However, application of external EFs as intense as +1.00 V/Å resulted in geometry instabilities and in difficulties in achieving the (tight) electronic convergence criteria employed.

**Figure 1:**
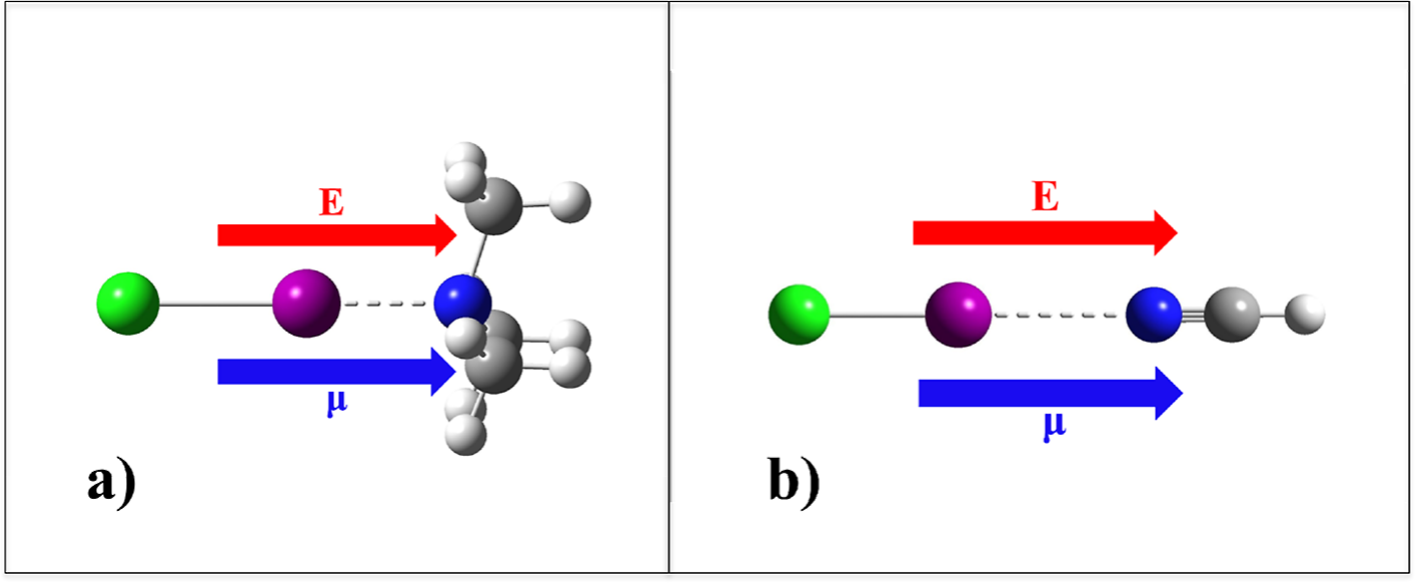
Halogen-bonded systems investigated in the current work: iodine chloride and trimethylamine (a), and iodine chloride and hydrogen cyanide (b). Atoms are color-coded as follows: carbon in gray, hydrogen in white, nitrogen in blue, iodine in violet, and chlorine in green. Arrows in red indicate the direction of the applied electric field when considered as “positive”, that is, concordant with the direction of the natural dipole moment (arrows in blue) of the investigated systems. Note, however, that directions opposite to that shown here have also been investigated in this work.

All calculations were carried out with either the quantum chemistry software Gaussian 16 (Rev. C.01)^75^ or PSI4 v1.9.1.^76^ In particular, geometry optimizations, electron densities, frequencies analysis, and molecular orbitals evaluation were conducted with Gaussian 16. The calculation of individual stabilizing and destabilizing energetic components was carried out with PSI4 using the SAPT (Symmetry-Adapted Perturbation Theory) method.^77^ More specifically, we adopted the SAPT2+(3)­δMP2 method, using the aug-cc-pVTZ basis set for light atoms and the aug-cc-pVTZ-PP basis set for the iodine atom. This combination of theory level and basis set is often referred to as the “gold standard” procedure for characterizing the various energy contributions underlying the behavior of noncovalent interactions.^78^ The molecular complexes considered for the SAPT method were optimized in Gaussian 16 using the *meta*-GGA M06-2X^60^ functional in combination with the def2-TZVP basis set.

## Results and Discussion

Although both complexes contain ICl and form a halogen bond (XB), they differ markedly. The XB complex with trimethylamine extends along all three Cartesian directions (Figure 1a), whereas the hydrogen-cyanide complex is linear and therefore essentially one-dimensional (Figure 1b). As it will be laid out later on, such a topological difference leads to measurable variations in the XB response to external static and homogeneous electric fields (EFs). Moreover, in the absence of any external perturbation also the XB lengths of the two systems are measurably different. As shown in Table 1, the XB lengths (i.e., the I···N bond distances) of the ClI···N­(CH_3_)_3_ complex range between a minimum value of 2.360 Å (CCSD/jorge-TZP-DKH) and a maximum value of 2.568 Å (B3LYP/def2-TZVP). Instead, as shown in Table 2, consistently larger intermolecular distances – on average on the order of ∼2.85 Å—are observed for the ClI···NCH complex, which suggests a weaker X-bonding in this system. Interestingly, while CCSD/jorge-TZP-DKH shows the shortest XB lengths in ClI···N­(CH_3_)_3_ (Table 1), the shortest I···N distance is obtained with the M06-2X/jorge-TZP-DKH method in ClI···NCH (Table 2).

**Table 1:** Equilibrium Halogen Bond (XB) Lengths, Defined as the I···N Bond Distance, for the ClI···N­(CH_3_)_3_ Complex

method	basis set	basis set for iodine	XB length (Å)
B3LYP	6-311++G(d,p)	LANL2DZ	2.549
B3LYP	6-311++G(d,p)	SDD	2.558
B3LYP	def2-TZVP	def2-TZVP	2.568
B3LYP	jorge-TZP-DKH	jorge-TZP-DKH	2.427
M06-2X	6-311++G(d,p)	LANL2DZ	2.457
M06-2X	6-311++G(d,p)	SDD	2.468
M06-2X	6-311++G(d,p)	def2-TZVP	2.470
M06-2X	aug-cc-pVTZ	aug-cc-pVTZ-PP	2.480
M06-2X	def2-TZVP	def2-TZVP	2.485
M06-2X	jorge-TZP-DKH	jorge-TZP-DKH	2.380
CCSD	def2-TZVP	def2-TZVP	2.517
CCSD	jorge-TZP-DKH	jorge-TZP-DKH	2.360

**Table 2:** Equilibrium Halogen Bond (XB) Lengths, Defined as the I···N Bond Distance, for the ClI···NCH Complex

method	basis set	XB length (Å)
M06-2X	aug-cc-pVTZ	2.829
M06-2X	def2-TZVP	2.841
M06-2X	jorge-TZP-DKH	2.815
CCSD	def2-TZVP	2.923
CCSD	jorge-TZP-DKH	2.872

Considering the ClI···N­(CH_3_)_3_ complex (Table 1), the B3LYP functional systematically gives sizably larger bond distances than the hybrid *meta*-GGA functional M06-2X, and CCSD calculations, both for the halogen and covalent I–Cl bonding (Figures S1 and S2 of the Supporting Information). This behavior, which has already been documented in the literature for other intermolecular interactions such as hydrogen (H−) bonds,^79^ can be attributed to the tendency of B3LYP to underestimate attractive forces of noncovalent nature, such as London dispersion.^80^ Remarkably, in the trimethylamine complex we observe a substantial difference in the equilibrium XB length (∼0.15 Å) between the CCSD/def2-TZVP and CCSD/jorge-TZP-DKH calculations (Table 1). This finding already suggests that the specific treatment of relativistic effects in the basis set can have a significant impact in iodine-containing systems.

Although, to the best of our knowledge, for the ClI···N­(CH_3_)_3_ complex no equilibrium XB lengths are available from the literature, some comparisons can be made for ClI···NCH. In particular, very accurate molecular geometries of simple dimeric XB systems were calculated at the CCSD­(T)/aug-cc-pVQZ theory level, including the ClI···NCH dimer, by Kozuch and Martin,^21^ who reported a XB distance of 2.816 Å. Such a value is in fairly good agreement with the estimate we get at the M06-2X/jorge-TZP-DKH theory level (i.e., 2.815 Å). This suggests a posteriori that the combination of the hybrid *meta*-GGA M06-2X functional and the jorge-TZP-DKH basis set performs well in predicting the equilibrium molecular geometry of this XB complex. Such an almost perfect agreement might partially reside in the high amount of exact exchange carried by the M06-2X DFT functional^21^ and in the quality of the basis set that embeds all-electron relativistic effects. As further support of this point, it is worth mentioning that also in this smaller X-bonded system a non-negligible equilibrium XB length difference of ∼0.05 Å is obtained at the CCSD level upon substituting the def2-TZVP basis set with the all-electron relativistic jorge-TZP-DKH one, as reported in Table 2.

Upon application of the EF, we observe a difference in the response of the XB lengths depending on the mutual orientation of the EF and the dipole moment vector of the complexes. Indeed, as shown in Figure 2, independently from the adopted combination of theory level and basis set, the application of a “positive” EF[Fn fn1] leads to a shortening of the intermolecular distance between the monomers involved both in the ClI···N­(CH_3_)_3_ (Figure 2a) and in the ClI···NCH (Figure 2b) complex.

**Figure 2:**
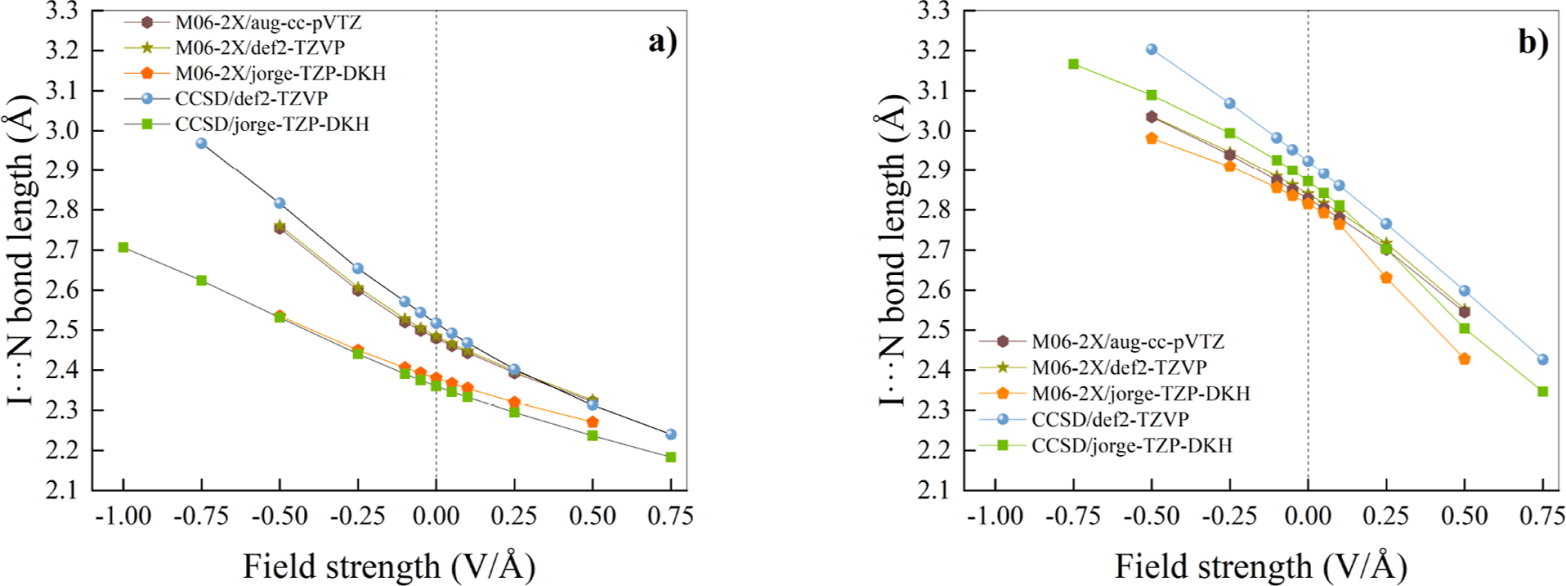
Halogen bond length, defined as the I···N intermolecular distance, as a function of the applied electric field strength for ClI···N­(CH_3_)_3_ (a) and ClI···NCH (b). Different combinations of levels of theory and basis sets are indicated in the respective legends. Vertical dotted lines mark the separation between the regions where a “negative” (left) and a “positive” (right) EF is applied.

Conversely, a “negative” EF produces in all cases an increase of the XB length. Such a circumstance is particularly interesting because, somehow surprisingly, similar CCSD computations of H-bonded systems exposed to external “negative” EFs previously investigated by our group^42^ were not yielding any (meta)­stable basin on the potential energy surface (PES) upon geometry relaxation. Instead, in H-bonded dimers, a field-induced complete reorientation of the molecular dipoles along the field axis was observed, a circumstance preventing any kind of analysis under those conditions. Similarly, in another important computational work,^46^ only the favorable arrangement between external EFs and some H-bonded and X-bonded dimers was considered. As shown in Figure 2a, the all-electron relativistic jorge-TZP-DKH basis set sizably improves at all EF strengths the M06-2X predictions of the XB length toward the CCSD/jorge-TZP-DKH values. Besides, especially for the ClI···N­(CH_3_)_3_ complex (Figure 2a) and the theory levels here investigated, it turns out that the impact of the basis set is more prominent than the specific usage of either *meta*-GGA hybrid functionals or explicitly correlated CCSD methods, as also stressed in the Supporting Information (see Figures S1 and S2).

However, when the linear ClI···NCH complex is considered, a less systematic performance trend is observed, with the CCSD/jorge-TZP-DKH data lying approximately in the middle between the CCSD/def2-TZVP and the M06-2X/jorge-TZP-DKH ones, as reported in Figure 2b. It is worth noticing the surprisingly linear trend recorded for the XB length as a function of the field only at the CCSD/def2-TZVP level, with an *R* ^2^ = 0.9972. While we were not capable of clarifying the reasons behind the out-of-trend behavior exhibited by some data points at +0.25 and +0.50 V/Å in Figure 2b, it appears clear from both panels of Figure 2 that the CCSD/def2-TZVP combination sizably overestimates the XB length and its field response in both iodine-containing complexes across all the EF regimes.

In addition to the field-induced shortening (elongation) of the intermolecular distance between the monomers of the two halogen-bonded complexes here investigated, also the intramolecular-covalent-bond of the ICl species is measurably sensitive to the field strength and direction, as shown in Figure 3. Again, the use of the def2-TZVP basis set exaggerates, at all theory levels, the I–Cl covalent bond length response to the EF, especially in the trimethylamine-containing complex (Figure 3a). Albeit the functional form of the curves is slightly different, by combining the information from Figures 2a and 3a, it is clear that in the ClI···N­(CH_3_)_3_ halogen-bonded dimer the response to the external field is, to a large extent, dictated by the field-induced elongation (contraction) of the I–Cl covalent bond. Conversely, in the ClI···NCH complex, we observe a much larger field-induced variation of the XB length (Figure 2b) with respect to the corresponding variation produced by the same field strength on the I–Cl covalent bond (Figure 3b). This indicates a different overall response to the field for this linear XB system. Moreover, while in ClI···N­(CH_3_)_3_ the curvature of the XB and I–Cl bond–length profiles is the same (see, for example, Figures 2a and 3a), an opposite curvature is observed for the corresponding quantities in ClI···NCH (Figure 2b vs Figure 3b). In fact, a negative concavity is observed for the curve depicting the XB length response of ClI···NCH to the external field across the different theory levels and basis sets tested (Figure 2b). Hence, such a system-specific different XB behavior appears to be uncorrelated to the employed level of theory.

**Figure 3:**
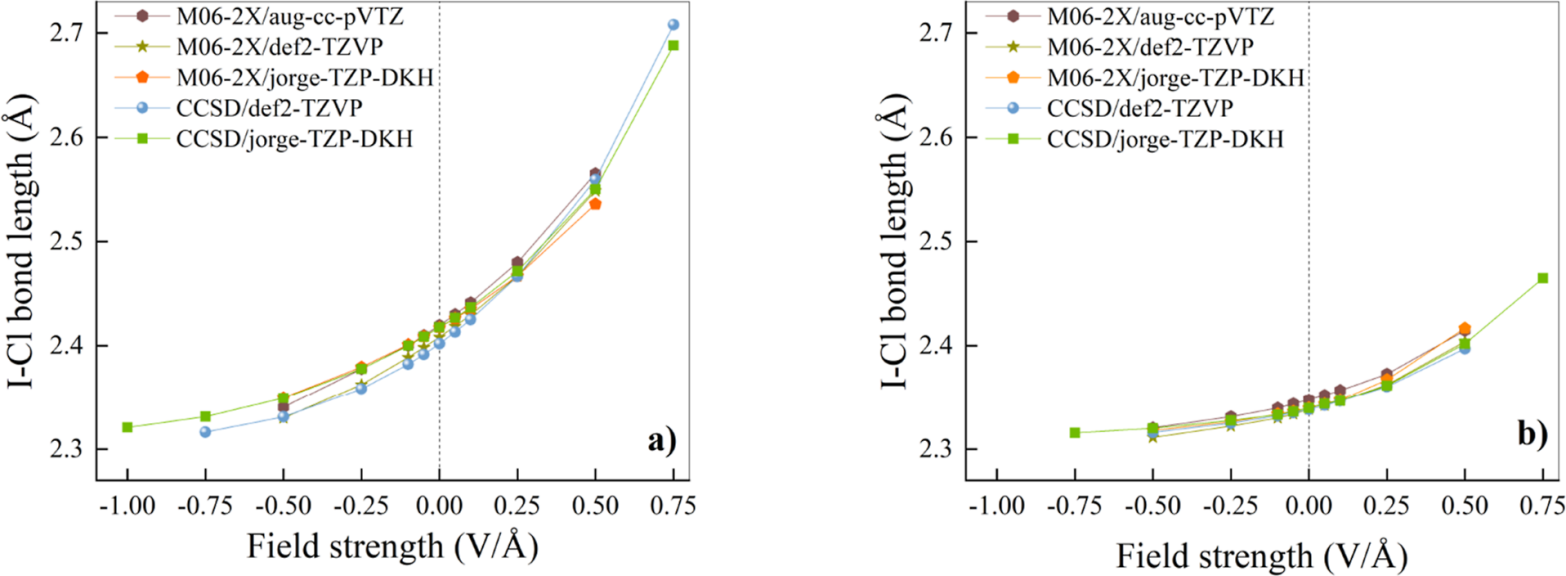
Iodine–chlorine bond length as a function of the applied EF strength for ClI···N­(CH_3_)_3_ (a) and ClI···NCH (b). Different combinations of levels of theory and basis sets are indicated in the respective legends. Vertical dotted lines mark the separation between the regions where a “negative” (left) and a “positive” (right) EF is applied.

All in all, our results indicate that both the XB and I–Cl length obtained at the CCSD level with the relativistic jorge-TZP-DKH basis set display a stronger resilience to changes in the applied field strength, pointing to a greater overall stability of the systems. Furthermore, though the usage of the def2-TZVP basis set tends to amplify the field effects on several geometrical properties, the progressive increase of the EF strength leads to measurable variations of the molecular geometries of the investigated XB dimers at all theory levels. Differently from common H-bonded dimers, where despite field-induced molecular rearrangements the H-bond length resulted to be quite insensitive to very strong fields even beyond +1.0 V/Å,^42^ the XB length turns out to be very sensitive to the application of low-to-moderate field regimes. Such a nontrivial finding suggests that the XB strength could be more susceptible than the H-bond one to electrostatic gradients, as also indicated elsewhere for positive-only EFs of ∼+0.25 V/Å.^46^

Accurate measurements of the strength of intermolecular interactions can be earned exploiting the vibrational Stark effect.^23^ Therefore, we have monitored the vibrational frequencies associated with the infrared-active stretching of the I–Cl bond donating the XB in the complexes here investigated. The calculation of CCSD stretching frequencies is certainly desirable but it is computationally demanding and it becomes prohibitive when adopting all-electron relativistic basis sets like the jorge-TZP-DKH one. Just to quantify the challenge, a single geometry relaxation of ClI···N­(CH_3_)_3_ at the CCSD/jorge-TZP-DKH level of theory implies the production of wave-function files of about 380 Gb each. Moreover, attempts of frequency calculations performed at the CCSD/jorge-TZP-DKH level have been aborted after weeks of calculations in large-memory computing nodes, while they failed due to memory lack on computing nodes equipped with less than 756 Gb of RAM. However, it is clearly visible from Figure 4 that the physical behavior of the Stark shift in ClI···N­(CH_3_)_3_ and ClI···NCH is essentially independent from the employed basis set. It is also quite independent from the theory level adopted, at least when the absolute value of the stretching frequency at a given field regime is rescaled by its zero-field value (see Figure S4 of the Supporting Information).

**Figure 4:**
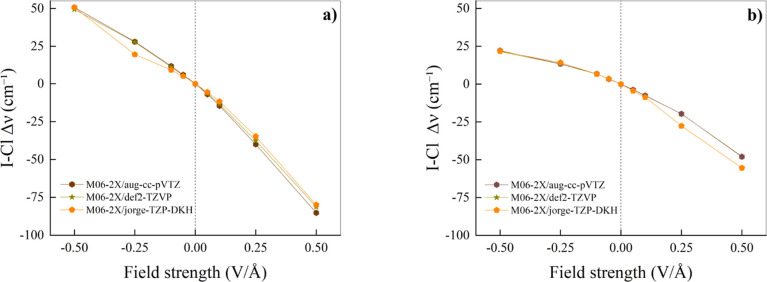
Stretching frequencies variations, relative to the zero-field case, of the iodine–chlorine stretching mode as a function of the field strength for ClI···N­(CH_3_)_3_ (a) and ClI···NCH (b). The M06-2X functional, along with different basis sets indicated in the respective legends, has been employed for this analysis. Vertical dotted lines mark the separation between the regions where a “negative” (left) and a “positive” (right) EF is applied.

Similar to H-bonded systems,^33,42,46^ the application of progressively stronger “positive” EFs leads to a red-shift of the vibrational frequency of the covalent bond donating the intermolecular bond, both in the ClI···N­(CH_3_)_3_ (Figure 4a) and in the ClI···NCH (Figure 4b) complex. Interestingly, both field-induced red-shifts rescaled by the respective zero-field frequency (see Footnote[Fn fn2]). At a field strength of +0.50 V/Å, $\Delta \mathrm{\nu ̅}/\mathrm{\nu ̅}=-80\;cm^{-1}/351\;cm^{-1}=-0.23$ in the trimethylamine complex and $\Delta \mathrm{\nu ̅}/\mathrm{\nu ̅}=-55\;cm^{-1}/408\;cm^{-1}=-0.14$ in the hydrogen-cyanide complex. These relative values of the Stark effect are at least 1 order of magnitude larger than the corresponding value determined on the O–H covalent bond donating the H-bond in the water dimer, namely, $\Delta \mathrm{\nu ̅}/\mathrm{\nu ̅}=-39\;cm^{-1}/3776\;cm^{-1}=-0.01$ (same field strength, M06-2X/aug-cc-pVQZ level).^81^ Such an evidence is indicative of a more pronounced relative field-induced strengthening of the XB in those systems with respect to that observed in H-bonded dimers.^42^ As displayed in Figure S4 of the Supporting Information, the fact that also non-relativistic basis sets predict comparable red-shifts indicates that such a behavior is genuine and general of the XB interaction and, to some extent, independent from the inclusion of relativistic effects in the basis set.

Interestingly, to the best of our knowledge, no data are available in the literature about the vibrational response of H-bonds to EFs oriented against the system’s molecular dipole, and hence leading—in principle—to an overall weakening of the intermolecular interactions. As far as our XB complexes are concerned, the application of “negative” EFs produces an overall blue-shift of the vibrational stretching frequencies, as shown in Figure 4. Clearly, this double-sided effect—leading to red/blue-shifts depending on the field direction—is fully consistent with the elongation/contraction of the I–Cl covalent bond previously discussed in Figure 3. Furthermore, as directly visible from the respective slopes of the vibrational Stark effect curves, the overall field-induced effect is again much more manifest in the bigger and more polarizable system containing the trimethylamine molecule with respect to the linear system containing the smaller hydrogen cyanide moiety. The higher responsiveness of ClI···N­(CH_3_)_3_ vs ClI···NCH can be primarily attributed to the larger zero-field α_*xx*_ term of the polarizability tensor (i.e., 71.929 au vs 35.838 au, respectively, at the M06-2X/jorge-TZP-DKH level).

To directly assess the field-induced strengthening/weakening of the intermolecular interactions, the binding energies of the two XB complexes have been determined up to the singles and doubles coupled cluster method along with perturbative triples (CCSD­(T)), by choosing the def2-TZVP and the jorge-TZP-DKH as basis sets. As alluded to earlier, to the best of our knowledge, no literature data are available for the ClI···N­(CH_3_)_3_ complex. On the other hand, accurate calculations were reported for the ClI···NCH system in the zero-field case. In fact, Kozuch and Martin^21^ report a binding energy of −6.31 kcal/mol at the CCSD­(T)/CBS level, by using non-relativistic reference basis sets up to aug-cc-pVQZ quality.^21^ As shown in Figure 5b, our zero-field estimates at the CCSD­(T) level, employing the small-core effective core potentials which embed scalar relativistic effects into the pseudopotential (def2-TZVP), and the all-electron relativistic jorge-TZP-DKH basis set, predict binding energies equal to −5.83 and −7.44 kcal/mol, respectively. This finding implies that the inclusion of all-electron relativistic effects sizably impacts the depth of the energy basin resulting from X-bonding involving the iodine element.

**Figure 5:**
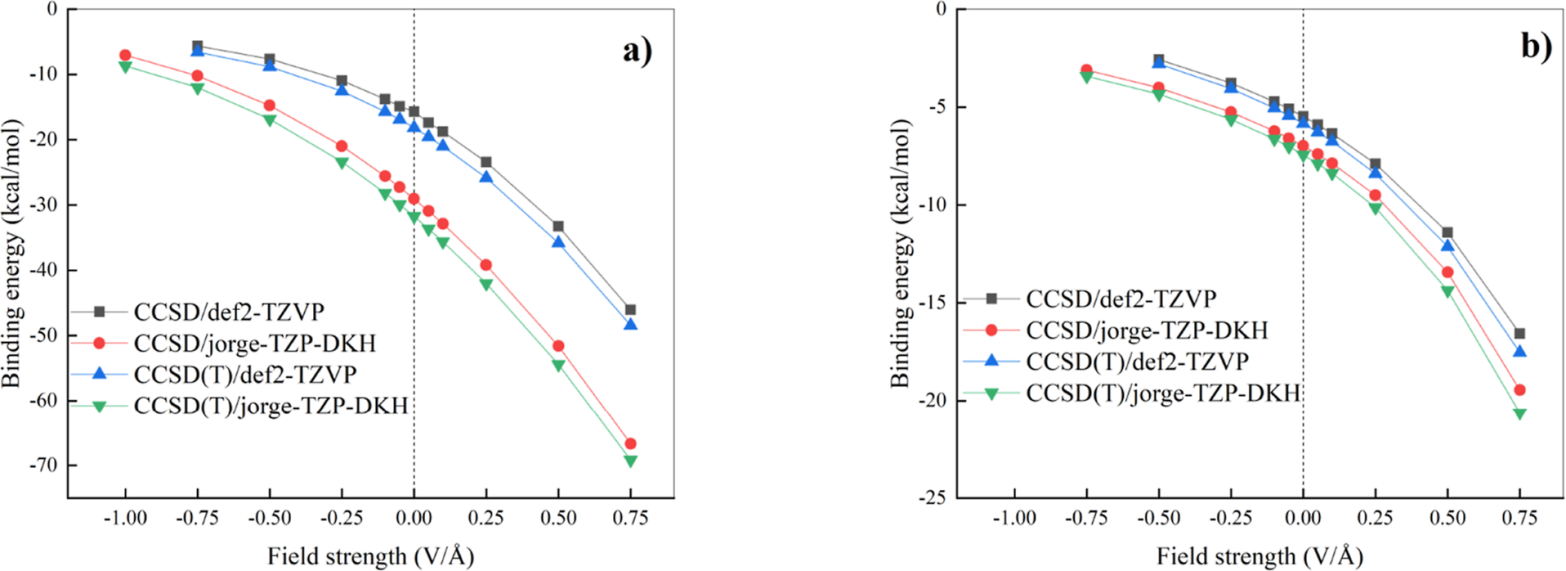
Binding energy of the ClI···N­(CH_3_)_3_ (a) and the ClI···NCH (b) complexes as a function of different EF intensities determined at the CCSD (black squares and red dots) and CCSD­(T) (up blue triangles and down green triangles) theory levels from the respective CCSD geometries with def2-TZVP and the all-electron relativistic jorge-TZP-DKH basis sets. Vertical dotted lines mark the separation between the regions where a “negative” (left) and a “positive” (right) EF is applied.

We obtain a surprisingly large difference in the zero-field binding energies of the ClI···N­(CH_3_)_3_ complex predicted by CCSD­(T)/def2-TZVP and CCSD­(T)/jorge-TZP-DKH. As displayed in Figure 5a, indeed, whereas a dissociation energy of 18.17 kcal/mol is required to separate the monomers when employing the def2-TZVP basis set, such a value almost doubles up to 31.73 kcal/mol in the presence of the all-electron relativistic jorge-TZP-DKH basis set. This striking difference obtained between the jorge-TZP-DKH and the def2-TZVP basis sets arises from the fundamentally different ways in which these basis sets treat relativistic effects and electron correlation in heavy atoms such as iodine. The jorge-TZP-DKH basis is an all-electron set explicitly optimized for use with the DKH Hamiltonian, thereby incorporating scalar relativistic corrections (i.e., mass–velocity and Darwin terms) directly in the valence region and providing a flexible description of the iodine orbitals. This ensures that subtle relativistic contractions of the 5s and 5p shells, as well as their consequences on valence stability and polarizability, are fully represented, which is particularly important for X-bonding where polarization and charge-transfer significantly contribute to the stabilization of complexes. By contrast, the def2-TZVP basis set employs a small-core relativistic effective core potential in which the core electrons are replaced by a core pseudopotential, so that only the valence region is described explicitly. While scalar relativistic effects are folded into the pseudopotential, the resulting valence basis is more compact and lacks the diffuse and polarization flexibility that XBs in principle demand. As a consequence, the def2-TZVP treatment underestimates both the degree of polarization and the charge-transfer stabilization, yielding markedly weaker binding compared to the more complete relativistic all-electron Jorge basis set.

The fundamental differences between basis sets discussed above have a measurable impact also on the behavior of the XBs under EFs. In fact, although less visible in the smaller and linear ClI···NCH system (Figure 5b), in the larger and more polarizable ClI···N­(CH_3_)_3_ complex (Figure 5a) a significantly larger slope testifies the response of the binding energy when the jorge-TZP-DKH basis set is employed. Moreover, the better description of field-induced polarization and charge transfer phenomena allows for the possibility to explore more extreme field regimes which are inaccessible via the def2-TZVP basis set because of the impossibility to achieve satisfactorily tight electronic convergence criteria for strengths beyond −0.50 V/Å in the ClI···NCH system, as displayed in Figure 5b. Nevertheless, all data prove that whereas “positive” EFs largely stabilize XBs, “negative” ones measurably destabilize the complexes here investigated, in line with previous analyses here presented on the Stark effect and on different molecular geometrical properties. Once again, the effects on the binding energy of these X-bonded complexes observed by applying external fields oriented along the molecular dipole moment appear to be much larger than those observed for H-bonded dimers.^42^ It is also evident that the more polarizable nonlinear complex shows similar variations of the binding energy under EFs of different sign (Figure 5a), while the binding energy of the linear ClI···NCH system is much more susceptible to “positive” rather than to “negative” EFs (Figure 5b). The field-induced (de)­stabilization of the investigated complexes described above is rooted in the fundamental behavior of local charges and electron densities. In an attempt of projecting the electron density onto the atomic basis set, we have determined the Mulliken charges for both XB systems as a function of the EF strength at the CCSD/jorge-TZP-DKH level of theory (Figure 6a,b). However, this kind of population analysis seemingly fails in correctly tracking the behavior of the charges for the large and highly polarizable ClI···N­(CH_3_)_3_ complex. In fact, as shown in Figure 6a, both the iodine and the nitrogen atoms lying on the XB appear to be quite insensitive to the field application, while the chlorine atom undergoes a prompt progressive increase of its own electron population as the field is augmented. Such an unexpected behavior—which is confirmed by reverting the field orientation—likely mirrors an overcounting of the electron fraction due to the nearby iodine atom under the field action.

**Figure 6:**
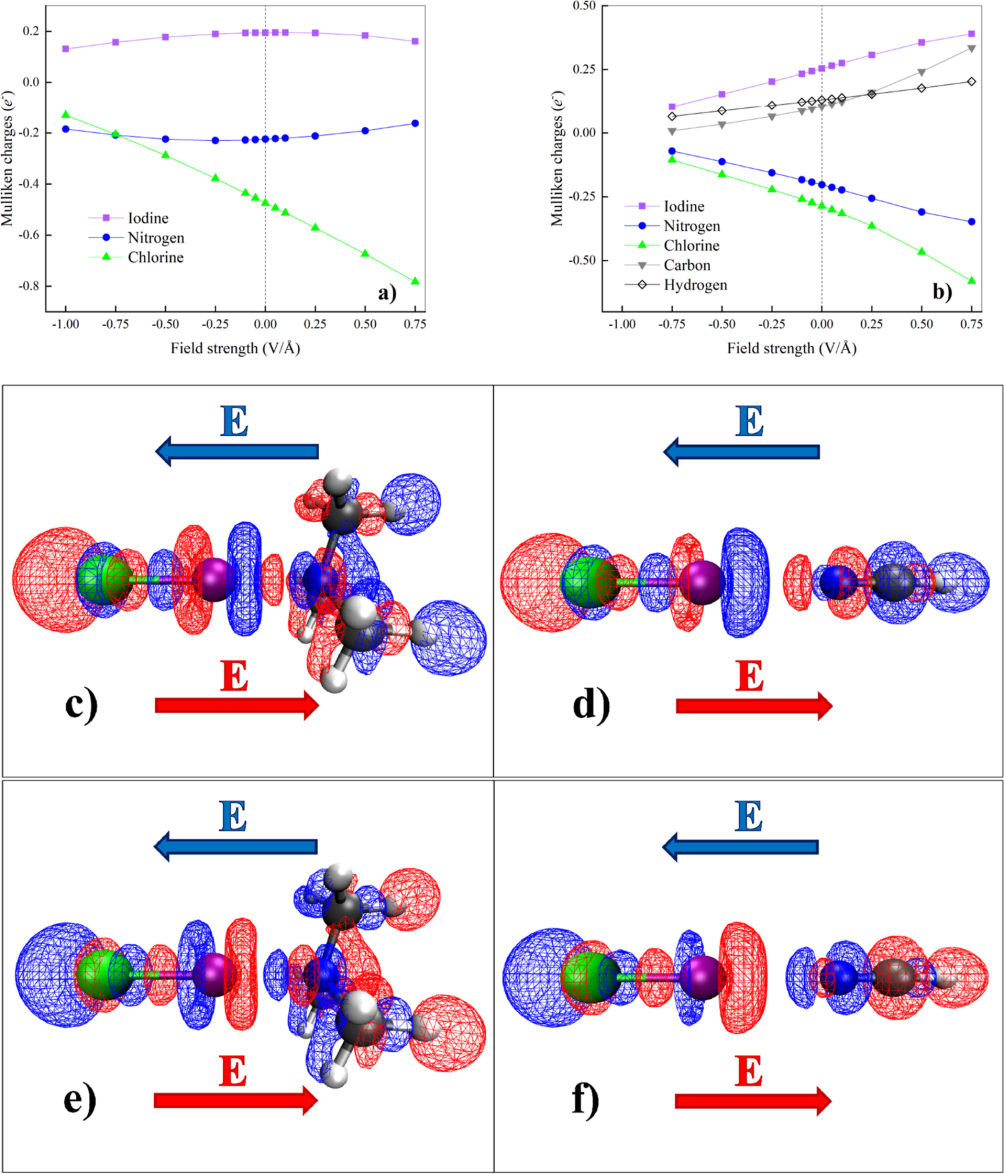
Mulliken charges localized on the iodine (purple squares), nitrogen (blue dots), and chlorine (green triangles) atoms of the ClI···N­(CH_3_)_3_ system (a) and for all the atoms composing the ClI···NCH complex (b) as a function of the EF strength. In the bottom panels, CCSD/jorge-TZP-DKH electron density differences (Δρ) for field strengths of −0.50 V/Å (blue isosurfaces) and +0.50 V/Å (red isosurfaces) determined by using the zero-field density and nuclear positions as a reference for the ClI···N­(CH_3_)_3_ (c,e) and ClI···NCH (d,f) complexes. For clarity, positive-only isocountours corresponding to Δρ = +0.001 au are displayed in (c,d) while negative-only isocountours corresponding to Δρ = – 0.001 au are displayed in (e,f).

As far as the ClI···NCH complex is concerned, a consistent physical picture can be earned from the Mulliken population analysis. As displayed in Figure 6b, the behavior of the iodine charge almost exactly anticorrelates with that of nitrogen. Although at a first sight this evidence might lead to conclude for a field-induced charge transfer between those elements under a “positive” EF, it has to be recognized that, surprisingly, the iodine atom loses charge while the nitrogen atom gets more electron rich upon increasing the field strength. This scenario clearly contradicts the physical displacement of the electron density induced by a “positive” field in our reference system. Closer inspection of the Mulliken charges of all the atoms composing the ClI···NCH complex solves this apparent puzzle. In fact, Figure 6b might be rationalized by considering that only local (i.e., within the same monomer) charge transfers occur at the explored field regimes. This way, whereas some charge moves from iodine to chlorine in I–Cl, some other is transferred from the CH group to nitrogen in the HCN molecule under the action of a “positive” EF. On the contrary, upon applying a “negative” field, an opposite charge displacement is observed within the same monomers, witnessing the EF capability of altering the covalency in X-bonded systems. Interestingly, within the Mulliken scheme, atomic charges tend to converge to 0 when the applied EF gets more and more “negative” (i.e., ∼−0.75 V/Å), as displayed in Figure 6b, a circumstance indicating some potential charge displacement saturation. Going beyond any electron density partitioning scheme, we report in the bottom panels of Figure 6 the electron density differences induced by the application of “positive” (red isocontours) and “negative” (blue isocontours) EFs. Isosurfaces of the CCSD/jorge-TZP-DKH electron density qualitatively confirms the previously described scenario, with the field capable of displacing sizable electron densities only in the proximity of the atoms and not in the internuclear region between the monomers, especially in the ClI···NCH complex (Figure 6d). However, likely due to hyperconjugation of the methyl groups of the trimethylamine^82^-containing system, the response of the C–H covalent bonds oriented along the field direction of the two complexes here investigated is opposite, as shown in Figure S3 of the Supporting Information. To further elucidate on the energy contributions stabilizing X-bonding in our systems under applied fields, we present below the results obtained from Symmetry-Adapted Perturbation Theory (SAPT). SAPT decomposes the interaction energy into four terms: electrostatics, London dispersion, induction, and exchange-repulsion. Figure 7 shows that the total interaction energy, similarly to the binding (dissociation) energy in Figure 5, becomes increasingly stabilizing as the “positive” applied field grows. In ClI···N­(CH_3_)_3_, it varies from −9.00 kcal/mol at −0.50 V/Å to −45.37 kcal/mol at +0.50 V/Å (Figure 7a), while in ClI···NCH it spans from −2.85 kcal/mol at −0.50 V/Å to −13.70 kcal/mol at +0.50 V/Å (Figure 7b). In contrast with the binding energy, the interaction energy considers only energy change upon formation of the complex, i.e., changes in the molecular geometries are not included in the interaction energy.

**Figure 7:**
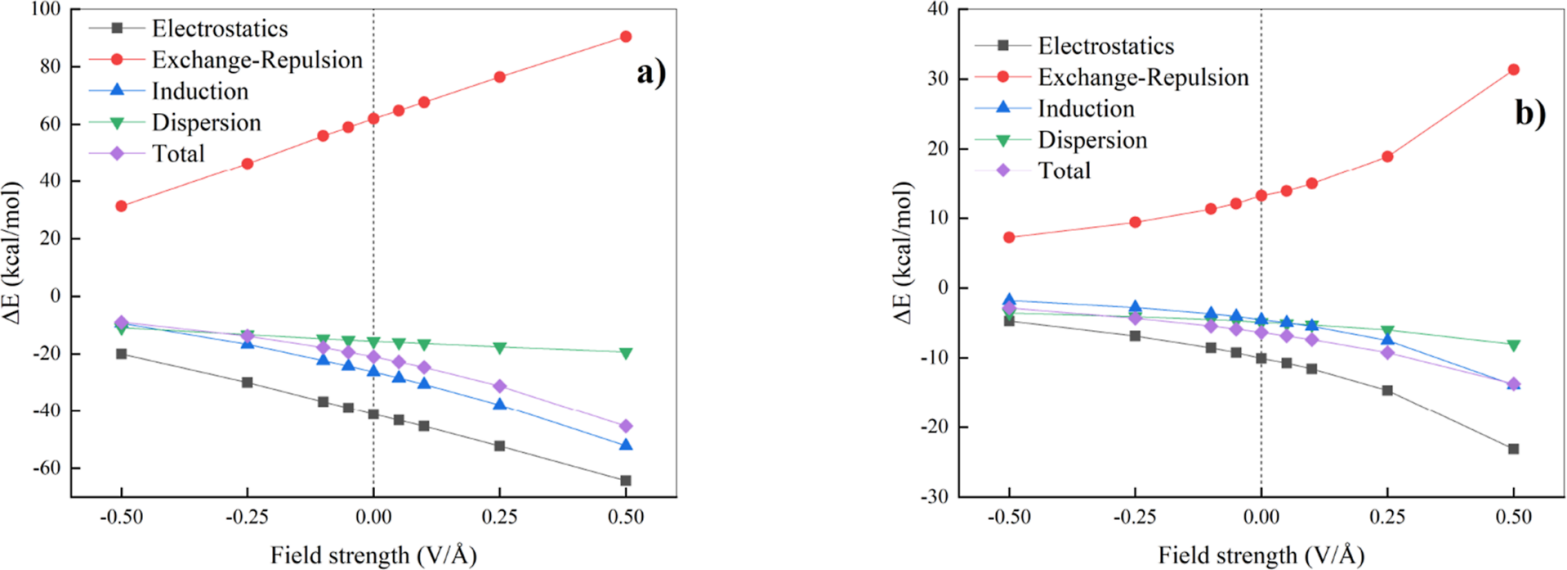
Contributions of individual energy components to the stability of the halogen bond between halogen molecule (ICl) and nitrogen-containing compound—in our case, trimethylamine (a) and hydrogen cyanide (b). Vertical dotted lines mark the separation between the regions where a “negative” (left) and a “positive” (right) EF is applied.

Furthermore, Figure 7 shows that electrostatics, induction, and London dispersion curves decrease under “positive” EFs (i.e., increase in their absolute value), leading to a strengthening of X-bonding. They are all stabilizing in the entire range of the investigated EF strengths, and all of these components contribute substantially to the stabilization of both XB complexes. However, the largest contributor to the stabilization of both X-bonded systems is electrostatics, which is followed by induction in the case of ClI···N­(CH_3_)_3_. For ClI···NCH, induction is stronger than London dispersion at larger “positive” fields. Then, exchange-repulsion (the only destabilizing term) increases with more “positive” EFs for both XB complexes, which is a result of shorter intermolecular distances (Figure 2). It is worth noting that the SAPT analysis indicates that both complexes considered here fulfill the IUPAC definition of an XB, as the stabilizing contributions are predominantly electrostatic in nature.^5^ However, Kolář et al. recognized an additional class of XB complexes in which London dispersion constitutes the dominant interaction-energy component.^17^ We also evaluated the relative contributions of the individual attractive interaction energy components (electrostatics, induction, and London dispersion) to the total stabilization energy given by the sum of the attractive components only. The corresponding data are reported in Figure 8, and show that electrostatics is highly dominant, i.e., its contribution to stabilization is around 50% for both XB complexes in the whole investigated range of EF strengths. At zero-field, specific terms contribute by 49%/32%/19% (electrostatics/induction/London dispersion) and by 52%/23%/25% (electrostatics/induction/London dispersion) to the total stabilization of ClI···N­(CH_3_)_3_ and ClI···NCH, respectively. While the contribution of electrostatics to the total stabilization slightly decreases with the more “positive” EFs in case of ClI···N­(CH_3_)_3_, it exhibits a concave behavior for ClI···NCH. Furthermore, while the importance of induction continuously increases with more “positive” EFs for both systems, the contribution of London dispersion to the total stabilization decreases almost symmetrically to the increase of induction. Interestingly, the dominant electrostatic component along the investigated range of EF intensities and a very similar trend in the increasing importance of the induction component and decreasing importance of London dispersion under “positive” EFs was observed at the same SAPT level for H-bonded complexes.^42^ Additionally, comparable trends but different contributions and magnitudes of induction and London dispersion were observed at the lower RI-SAPT0/aug-cc-pVDZ level of theory by Tokatlı et al., who investigated C-X···π halogen bonds under external EFs, using similar field intensities as those considered here.^83^

**Figure 8:**
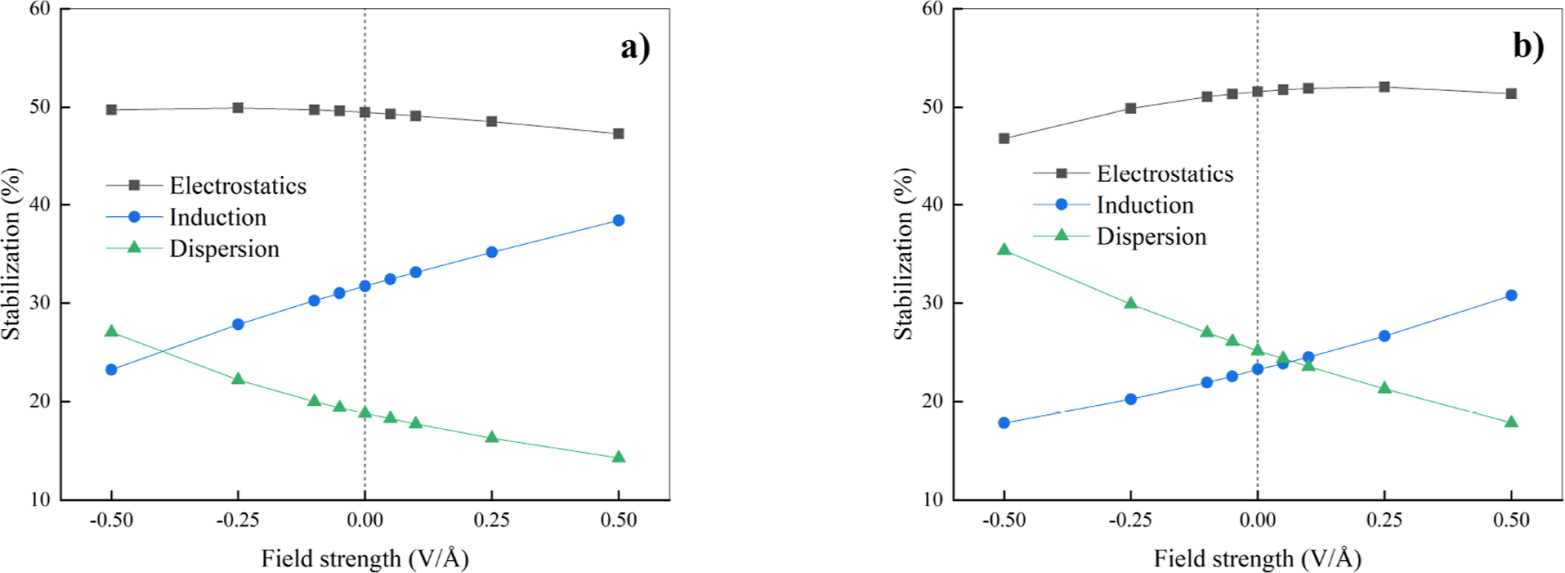
Percentage of energy contributions of individual components positively contributing to the stability of the halogen bond between halogen molecule (ICl) and nitrogen-containing compound - in our case, trimethylamine (a) and hydrogen cyanide (b). Vertical dotted lines mark the separation between the regions where a “negative” (left) and a “positive” (right) EF is applied.

## Conclusions

In this work, we have investigated the response of iodine-containing halogen bonds (XBs) to oriented external static and homogeneous electric fields (EFs) by combining wave-function methods up to the CCSD­(T) level, Density Functional Theory (DFT), relativistic and non-relativistic basis sets, and Symmetry-Adapted Perturbation Theory (SAPT) analyses.

The results reveal that the description of XB in heavy-atom systems is strongly influenced by the treatment of relativistic effects, both in the absence of an external field and under field application. In particular, the all-electron relativistic jorge-TZP-DKH basis set predicts significantly shorter bond lengths and substantially stronger binding energies than the basis sets based on effective-core-potential approaches such as def2-TZVP, which underscores the need for explicit relativistic treatments. The application of external EFs produces a pronounced modulation of the XB strength, with “positive” fields aligned with the complex dipole moment leading to substantial XB stabilization and “negative” fields inducing marked destabilization. Benchmarking different exchange–correlation DFT functionals against CCSD data proves the accuracy of M06-2X for X-bonded systems exposed to EFs, while also showing that the explicit treatment of relativistic effects in the basis set has a significantly larger influence than the specific choice of the DFT functional. Furthermore, the responsiveness to external fields exhibited by the two investigated complexes (ClI···N­(CH_3_)_3_, ClI···NCH) is measurably more intense than that observed in H-bonded systems.^42^ This is confirmed by evaluation of CCSD­(T)/jorge-TZP-DKH binding energies and by the large field-induced vibrational Stark shifts associated with the I–Cl stretching mode.

SAPT analyses further clarify that electrostatics provides the dominant stabilizing component at all field strengths, while induction plays an increasingly relevant role under stronger perturbations in both XB complexes. Overall, our findings indicate that XBs are highly sensitive to both relativistic effects and external electrostatic perturbations. By providing accurate quantum chemistry benchmarks including relativistic effects, this study highlights the importance of quantum-accurate approaches for iodine-based interactions and points to future opportunities for exploiting field-responsive X-bonding in catalysis, molecular recognition, and materials design.

## Supplementary Material



## Footnotes

a See Figure 1 and the Methods section for the definition of the reference system adopted in this study and for the specific convention of the EF orientation.

b For simplicity we approximate the ICl oscillating bond in the complexes as an oscillatory bond of a diatomic molecule (i.e., we discard any other recoil displacement happening in the two complexes during the normal motion). Then we can write ω = (*k*/μ)^1/2^ where *k* is the harmonic force constant of the bond, and μ = *m*_Cl_ *m*_I_/(*m*_Cl_ + *m*_I_) is the reduced mass. The change in the vibrational angular frequency for a change in the force constant (given by the applied field) can be computed considering that $\partial \omega /\partial k=1/\left(2\sqrt{\mu k}\right)$ . Therefore, at first order in Δ*k*, we have $\Delta \omega =\Delta k/\left(2\sqrt{\mu k}\right)$ . Hence, Δω (or Δν̅) is not a direct proxy of Δ*k* because it contains a residual dependence on the reduced mass, as well as the unperturbed value of the force constant, *k*. A better quantity to examine should be $\Delta \mathrm{\nu ̅}/\mathrm{\nu ̅}=\Delta \omega /\omega =\frac{1}{2}\Delta k/k$ , which is directly proportional to the relative change in the force constant.

## Notes

Extensive benchmark of the different theory levels and basis sets employed, potential energies, dipole moments, and molecular orbitals calculations (PDF)
