# Supplementary material for: Halogen Bonds under Electric Field with Quantum Accuracy and Relativistic Basis Sets
Source: J Phys Chem A. 2026 Jan 4;130(2):522–33. doi: 10.1021/acs.jpca.5c08038 (PMC12814542; doi:10.1021/acs.jpca.5c08038)
Supplement: Supplementary file 1 [file jp5c08038_si_001.pdf]

Supporting Information

Halogen Bonds under Electric Field with Quantum Accuracy and Relativistic Basis Sets

Gabriele Ottaná,[†,‡] Simona Mastronardo,[†,‡] Petr Eminger,[¶,§,‡] Klaudia Mráziková,[‖,¶,‡] Sebastiano Trusso,[‖] Franz Saija,[‖] Martin Ferus,[¶] Luigi Monsú Scolaro,[†] Jing Xie,[⊥] Matteo Tommasini,[#] and Giuseppe Cassone[*,‖]

†*Department of Chemical, Biological, Pharmaceutical, and Environmental Sciences, University of Messina, 31 Viale F. Stagno d'Alcontres, 98166 Messina (Italy)*

‡*These authors contributed equally*

¶*J. Heyrovský Institute of Physical Chemistry, Academy of Sciences of the Czech Republic Dolejškova 3, CZ18223, Prague 8 (Czech Republic)*

§*Faculty of Science, Department of Physical and Macromolecular Chemistry, Charles University in Prague,12840 Prague (Czech Republic)*

‖*Institute for Chemical-Physical Processes, National Research Council of Italy (CNR-IPCF), V. Stagno d'Alcontres 37, 98158 Messina (Italy)*

⊥ *Ministry of Education Key Laboratory of Cluster Science, Beijing Key Laboratory of Photoelectronic/Electrophotonic Conversion Materials, School of Chemistry and Chemical Engineering, Beijing Institute of Technology, Beijing 100081 (P. R. China)*

#*Dipartimento di Chimica, Materiali e Ing. Chimica "G. Natta", Politecnico di Milano, Piazza Leonardo da Vinci, 32, 20133 Milano (Italy)*

E-mail: giuseppe.cassone@cnr.it

Theory levels and basis sets benchmark on ClI$\cdots$N(CH_3)$_3$

In the current Supporting Information (SI) we discuss the results concerning benchmark analyses isolating the impact of the Density Functional Theory (DFT) exchange-correlation functional choice and the influence of the implementation of specific relativistic contributions focused on the ClI$\cdots$N(CH_3)$_3$ system, along with additional analyses on the two halogen (X-)bonded systems investigated in the main text, including dipole moments and molecular orbitals.

Fig. S1 shows the XB lengths of the ClI$\cdots$N(CH_3)$_3$ X-bonded complex exposed to both "positive" and "negative" electric fields (EFs) at different levels of theory. It turns out that calculations carried out with the jorge-TZP-DKH basis set, Fig. S1-d, give the lowest values with respect to remainder basis sets that do not account for all-electron relativistic effects (*i.e.,* 6-311++G(d,p), SDD, LANL2DZ, def2-TZVP), both when employing the hybrid B3LYP (Fig. S1-a) and the hybrid meta-GGA M06-2X functional (Fig. S1-b). As mentioned in the main text, Fig. S1-d shows that the B3LYP level of theory overestimates the XB length. In fact, while the equilibrium value falls at 2.427 Å with the B3LYP functional at zero field, the predicted XB distances by the M06-2X and CCSD theory levels locate at 2.380 Å and 2.360 Å, respectively. Furthermore, we observe a systematic difference between the values obtained through the B3LYP and the M06-2X exchange-correlation functionals combined with either LANL2DZ or SDD basis sets, with LANL2DZ exhibiting a mean difference of 0.094 ± 0.001 Å and SDD of 0.089 ± 0.001 Å between the two theory levels.

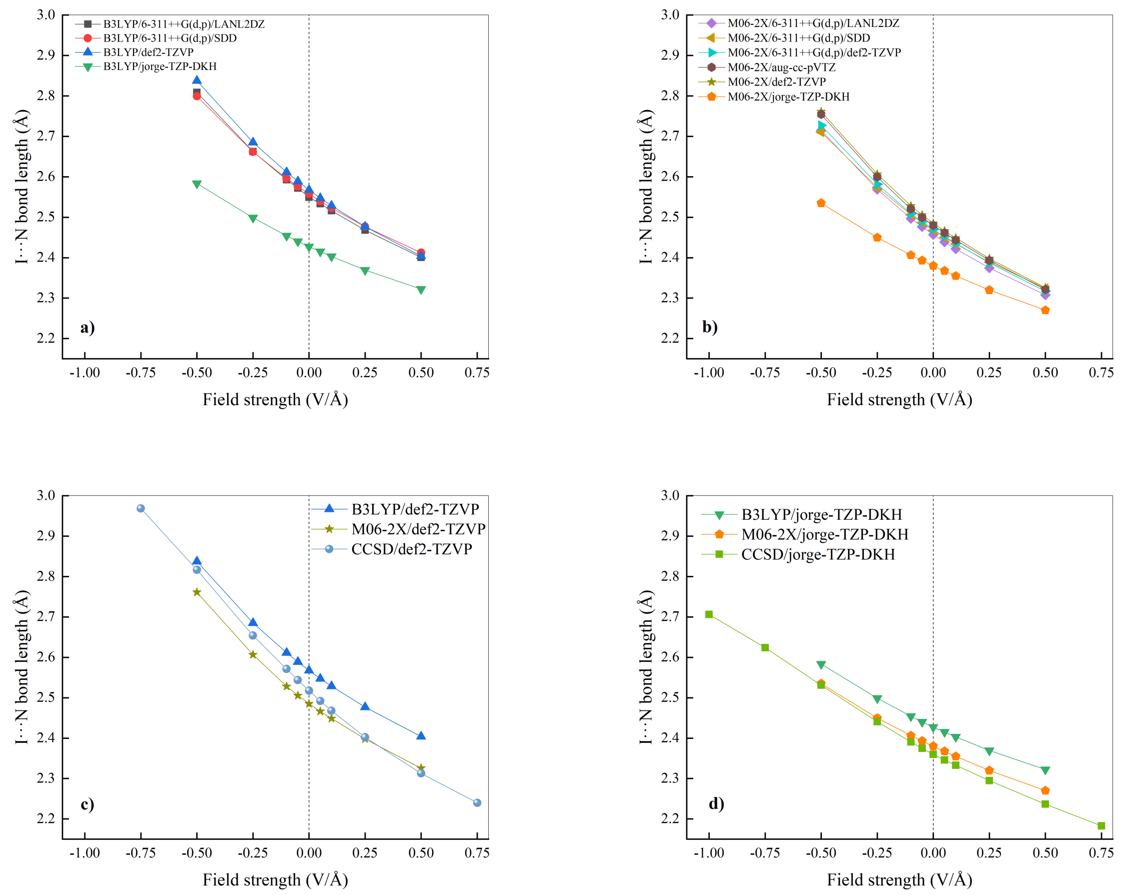

Figure S1: Halogen bond length as a function of the applied EF strength for the ClI···N$(CH_3)_3$ complex for all B3LYP calculations (a), for all M06-2X calculations (b), for all calculations exectuted with the def2-TZVP basis set (c) and for all those employing the all-electron relativistic jorge-TZP-DKH basis set (d). Vertical dotted lines mark the separation between the regions where a "negative" (left) and a "positive" (right) EF is applied. The 6-311++G(d,p)/LANL2DZ, 6-311++G(d,p)/SDD and 6-311++G(d,p)/def2-TZVP notation indicates that LANL2DZ, SDD and def2-TZVP basis sets were used for iodine while the Pople's 6-311++G(d,p) basis set was used for the rest of the atoms.

Interestingly, Fig S1-c shows that the M06-2X level of theory gives lower XB lengths than the CCSD one across almost the entire field range, when both paired with the def2-TZVP basis set. Such a trend is not recorded when employing the jorge-TZP-DKH basis set (Fig S1-d), although for more "negative" EF strengths M06-2X and CCSD calculations overlap each other. The XB lengths determined at the CCSD/def2-TZVP and CCSD/jorge-TZP-DKH levels of theory have different sensitiveness toward the applied EF, as shown in Fig. S1-c and Fig. S1-d. In particular, whereas in the absence of the EF the XB length difference is 0.157 Å, in presence of a "negative" EF such a difference increases up to a maximum of

0.345 Å (-0.75 V/Å), implying that the gap between the values increased by $\sim$ 119% from its initial value. On the contrary, when the ClI$\cdots$N$(CH_3)_3$ complex is exposed to "positive" EFs, the XB length decreases to a minimum value equal to 0.057 Å (+0.75 V/Å), accounting for a difference reduction of $\sim -64\%$.

I-Cl covalent bond lengths are susceptible to externally applied EFs. However, as partially expected, we do observe completely different trends compared to the XB lengths ones as a function of the field strength. First, Fig. S2-a and S2-b reveal that calculations employing the all-electron relativistic jorge-TZP-DKH basis set do not give the lowest values, with the def2-TZVP ones predicting the shortest I-Cl covalent bonds at zero-field conditions and under "negative" field intensities. On the other hand, for mid "positive" EF strengths, they tend to overlap each other, especially when employing the B3LYP functional (Fig. S2-a). It is interesting to notice that, among the various investigated basis sets, the I-Cl bond lengths obtained via the calculations executed with the LANL2DZ and SDD ones are the largest, as shown in Fig. S2-a and S2-b. B3LYP overestimates the I-Cl bond length not only in combination with the LANL2DZ and SDD basis sest, but also with def2-TZVP and jorge-TZP-DKH, as shown in Fig. S2-c and Fig. S2-d. Furthermore, it is noticeable that the I-Cl covalent bond lengths determined at the CCSD and M06-2X levels of theory overlap over the entire investigated EF strength range. Clearly, compared to the I$\cdots$N distances, there are smaller differences between the CCSD/def2-TZVP and CCSD/jorge-TZP-DKH curves. However, the def2-TZVP calculations exhibit lower values than jorge-TZP-DKH except for +0.50 V/Å and +0.75 V/Å, which, again, indicates a slightly different susceptibility toward the EF.

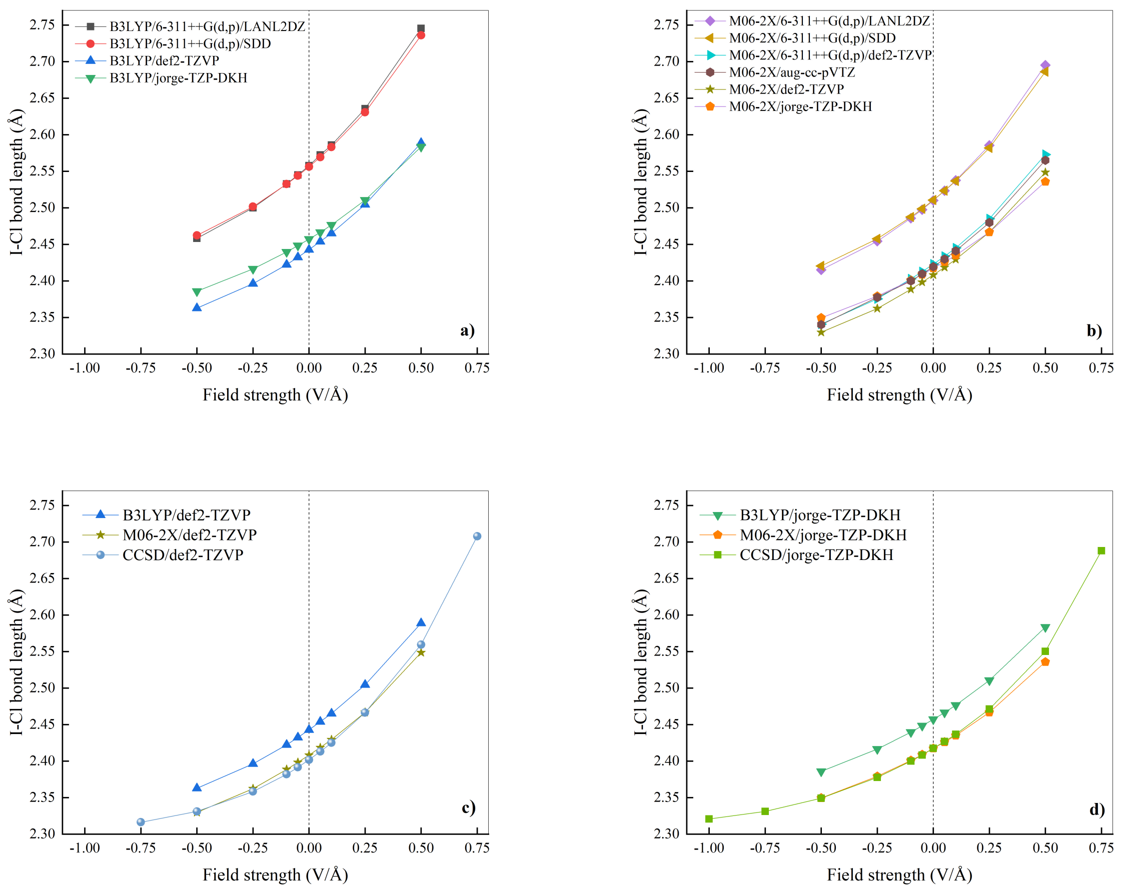

Figure S2: I-Cl covalent bond length as a function of the applied EF strength for the ClI$\cdots$N(CH_3)$_3$ complex for all B3LYP calculations (a), for all M06-2X calculations (b), for all calculations exectuted with the def2-TZVP basis set (c) and for all those employing the all-electron relativistic jorge-TZP-DKH basis set (d). Vertical dotted lines mark the separation between the regions where a "negative" (left) and a "positive" (right) EF is applied. The 6-311++G(d,p)/LANL2DZ, 6-311++G(d,p)/SDD and 6-311++G(d,p)/def2-TZVP notation indicates that LANL2DZ, SDD and def2-TZVP basis sets were used for iodine while the Pople's 6-311++G(d,p) basis set was used for the rest of the atoms.

An interesting finding, due to the same behavior of the properties analyzed for both systems, is the different trend in the C-H bond lengths of the two systems, as shown in Fig. S3. Apparently, as the applied external EF strength increases, the C-H covalent bond in the hydrogen-cyanide system is stretching whilst the C-H bonds of trimethylamine parallel to the external EF are contracting. This is linked to the different state of hybridization of the C atom in both systems. In the former system, C is *sp*, which makes the C-H bond 50% p-character and 50% s-character, whilst in the latter system C is sp^3 and the C-H bond has a 75% p-character and 25% s-character. This means that HCN has a C atom which attracts

mostly the electron density, as shown in Fig. 6 of the main text, implying a lower stability. This does not occur for the C-H bonds in trimethylamine due to the different hybridization state, which makes it less electrophile, and to the hyperconjugation, making the methyl groups more stable under "positive" EFs.

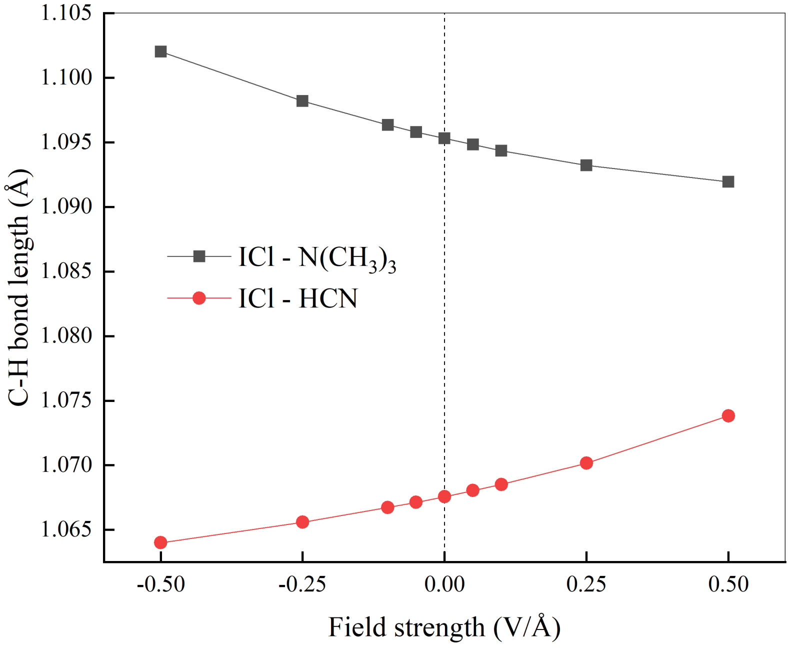

Figure S3: C-H covalent bond lengths for the two X-bonded systems investigated in this work (see legend) as a function of the applied external EF strength at the M06-2X/jorge-TZP-DKH level. The C-H bonds considered in the case of the complex containing trimethylamine are the three oriented preferentially toward the EF direction. Incidentally, their three lengths are exactly equal among each other upon optimization. Vertical dotted lines mark the separation between the regions where a "negative" (left) and a "positive" (right) EF is applied.

Fig. S4 shows the I-Cl stretching frequencies – relative to the zero-field case – at different EF strengths and for the different levels of theory employed in the current work. As detailed in the main text, we do not provide the corresponding CCSD values because of the prohibitive computational cost associated with these calculations with these systems and relativistic basis sets. Moreover, a difficult-to-explain outlier for an EF strength of -0.25 V/Å is recorded at the M06-2X/jorge-TZP-DKH theory level.

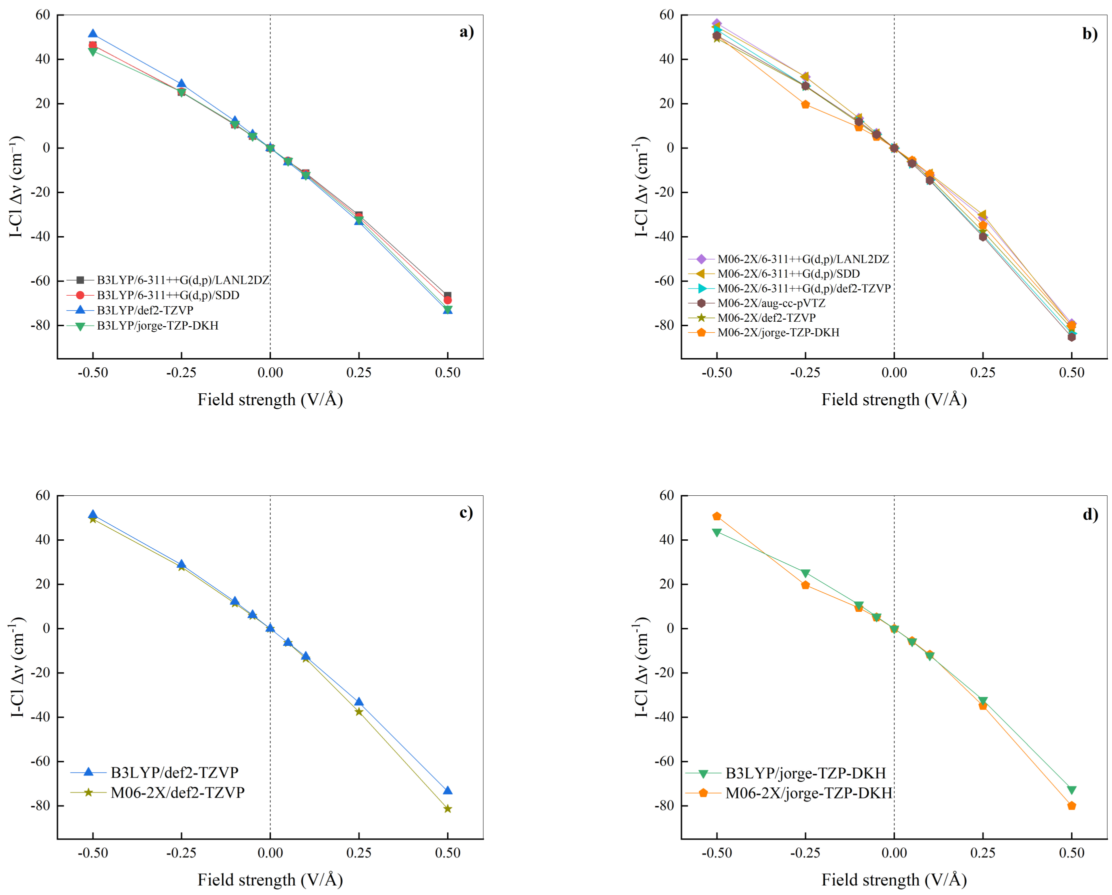

Figure S4: Stretching frequencies variations, relative to the zero-field case, of the iodine-chlorine stretching mode as a function of the applied EF strength for the ClI$\cdots$N$(CH_3)_3$ complex for all B3LYP calculations (a), for all M06-2X calculations (b), for all calculations exectuted with the def2-TZVP basis set (c) and for all those employing the all-electron relativistic jorge-TZP-DKH basis set (d). Vertical dotted lines mark the separation between the regions where a “negative” (left) and a “positive” (right) EF is applied. The 6-311++G(d,p)/LANL2DZ, 6-311++G(d,p)/SDD and 6-311++G(d,p)/def2-TZVP notation indicates that LANL2DZ, SDD and def2-TZVP basis sets were used for iodine while the Pople’s 6-311++G(d,p) basis set was used for the rest of the atoms.

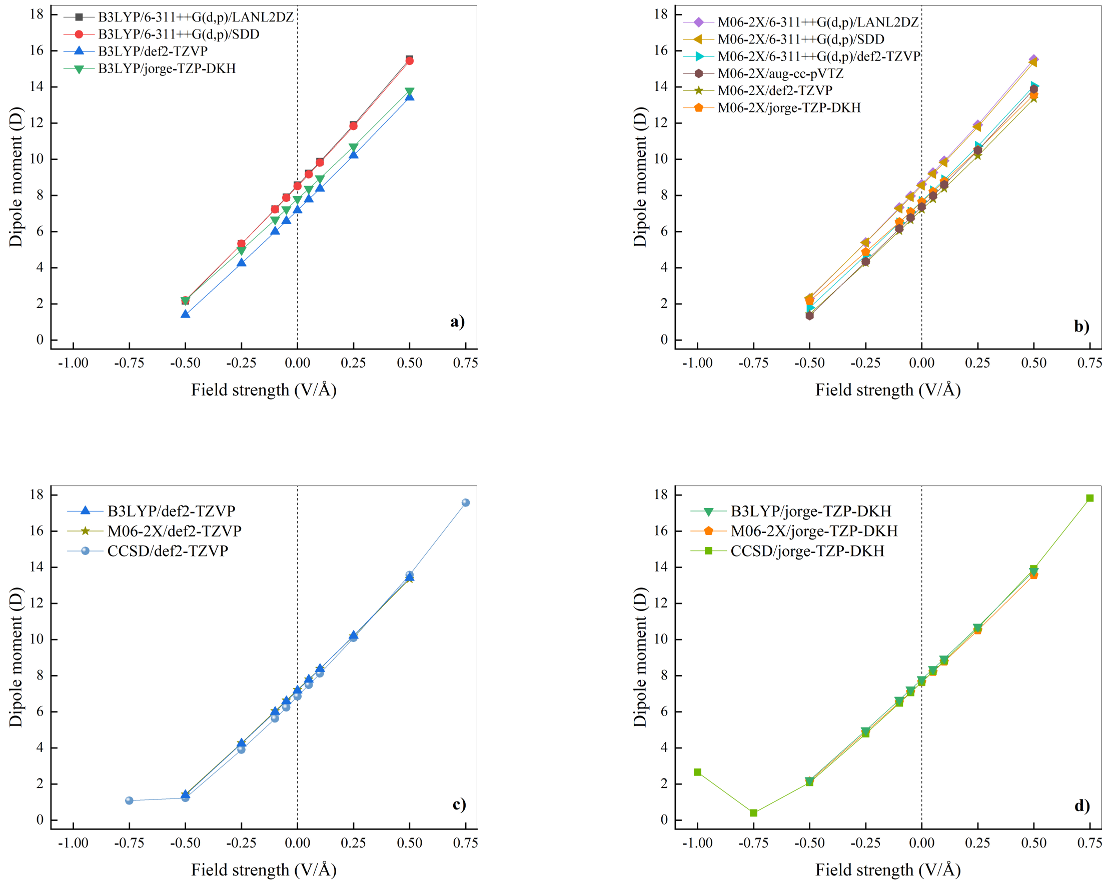

Figure S5: Dipole moment as a function of the applied EF strength for the ClI$\cdots$N$(CH_3)_3$ complex for all B3LYP calculations (a), for all M06-2X calculations (b), for all calculations exectuted with the def2-TZVP basis set (c) and for all those employing the all-electron relativistic jorge-TZP-DKH basis set (d). Vertical dotted lines mark the separation between the regions where a "negative" (left) and a "positive" (right) EF is applied. The 6-311++G(d,p)/LANL2DZ, 6-311++G(d,p)/SDD and 6-311++G(d,p)/def2-TZVP notation indicates that LANL2DZ, SDD and def2-TZVP basis sets were used for iodine while the Pople's 6-311++G(d,p) basis set was used for the rest of the atoms.

Potential energies and dipole moments

A central role in the response of molecular systems to external EFs is certainly played by the potential energy, which is mostly governed by the behavior of the dipole moment. Such quantities are shown in Figs. S6 and S7 for the systems containing trimethylamine and the system bearing hydrogen cyanide, respectively.

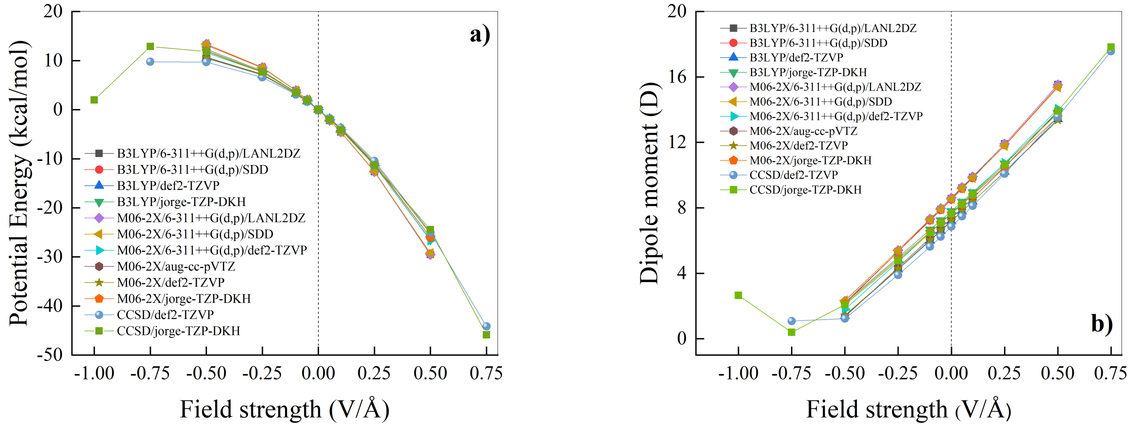

Figure S6: Potential energies relative to the zero-field case (a) and dipole moments (b) both as a function of the applied EF strength for different levels of chemical theory and basis sets, as indicated in the legends, for the ClI···N(CH_3)$_3$ complex. Vertical dotted lines mark the separation between the regions where a "negative" (left) and a "positive" (right) EF is applied.

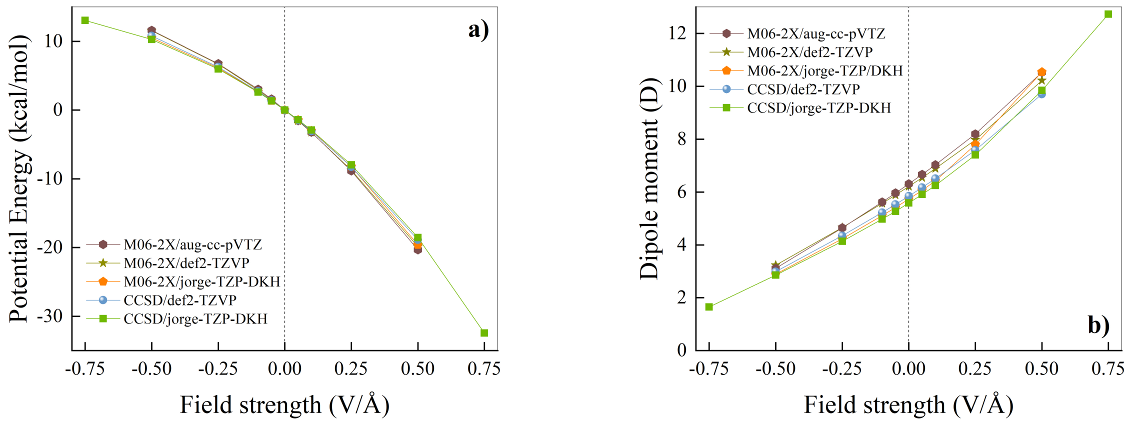

Figure S7: Potential energies relative to the zero-field case (a) and dipole moments (b) both as a function of the applied EF strength for different levels of chemical theory and basis sets, as indicated in the legends, for the ClI···NCH complex. Vertical dotted lines mark the separation between the regions where a "negative" (left) and a "positive" (right) EF is applied.

From Figs. S6 and S7 there is further confirmation of the strengthening of X-bonding induced by the EF when the direction of application is concordant with the dipole moment. In particular, both systems are subjected to a larger stabilization than in the case where the field is absent and, as a natural consequence, the total dipole moment increases under the action of the field. This evidence follows from the global Hamiltonian definition. Denoting as $\hat{H}_0$ the Hamiltonian of the system in the absence of EF, the Hamiltonian of the perturbed system is $\hat{H} = \hat{H}_0 - \hat{\boldsymbol{\mu}} \cdot \mathbf{F}$, where $\hat{\boldsymbol{\mu}}$ is the dipole moment of the system and $\mathbf{F}$ is the EF

vector. The expectation value of the dipole moment is closely related to the energy change of the ground state according to the relationship $\Delta E = \langle \hat{H}_0 \rangle - \langle \hat{\boldsymbol{\mu}} \rangle \cdot \mathbf{F}$, which can also be written as $\frac{d\Delta E}{d\mathbf{F}} = -\langle \hat{\boldsymbol{\mu}} \rangle \cdot \mathbf{u}_F$, where $\mathbf{u}_F$ is the versor along the EF direction.

From these equations, it follows that the application of an external EF opposite to the direction of the natural dipole moment of the system induces destabilization (*i.e.*, a relative increase of the total energy of the system). When the strength of the EF oriented in the opposite direction of the dipole moment vector becomes particularly intense, up to the point that the expected value of the dipole moment cancels out, a stationary point on the potential energy surface is reached, as visible from the −0.75 V/Å data point at the CCSD/jorge-TZP-DKH level of Fig. S6-a. Beyond that point, in artificially extreme regimes, a flipping of the electron dipole moment induced by the EF can be observed, as visible from the data point at −1.0 V/Å in Fig. S6-b. Such field-induced dipole moment reversal was also observed in simpler H-bonded dimers, but at more extreme field regimes.[1]

Nevertheless, in the calculations carried out on the ClI$\cdots$N$(CH_3)_3$ complex, it can be observed that there is a more linear correlation between the dipole moment magnitude and the EF strength, while there is a slight curvature in the response of the system containing hydrogen cyanide, as visible from a comparison of Figs. S6-b and S7-b. This could be due to the fact that trimethylamine, as a monomer in the zero-field regime, exhibits from experiments a relatively low dipole moment of 0.61 D, whereas that of hydrogen cyanide is almost 5 times larger (*i.e.*, 2.98 D).[2] Our simulations, performed on the individual monomers at the CCSD/jorge-TZP-DKH level, return dipole moment values of 0.64 D and 3.01 D for N$(CH_3)_3$ and HCN, respectively. The relative percent error associated with these measurements compared to the experimental data is then ∼4.7% and ∼1.1%, respectively, supporting the fairly good accuracy of the computational method here employed as a reference. Thereby, the measured difference in the dipole moments magnitude could underlie the differences observed in the responses to the applied EF and the emergence of nonlinear effects in the case of the X-bonded system containing the hydrogen cyanide moiety. In addition, the fact that this

system develops one-dimensionally along the direction of field application should be considered, a circumstance that could amplify more complex orbital phenomena along this axis. In order to rationalize this evidence as well, it is appropriate to visualize the molecular orbitals of the investigated systems under various applied field conditions, as reported in the next section.

Molecular orbitals

The influence of the EF on the electronic structure is further evidenced by the trend of the band gap, *i.e.*, the difference in the energies characterizing the HOMO and LUMO molecular orbitals, shown in Fig. S8. This analysis was carried out at the highest level of theory adopted in this work, namely the CCSD/jorge-TZP-DKH level.

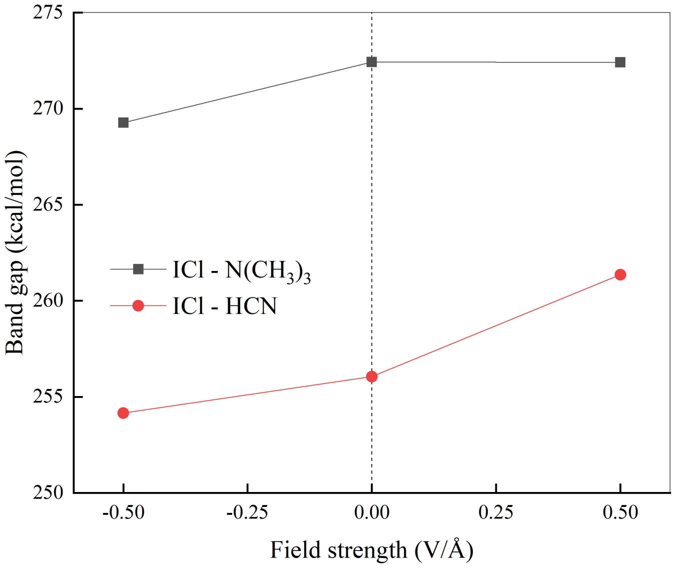

Figure S8: Energy difference between the LUMO and HOMO visualized in Figs. S9 and S10 for the two X-bonded systems investigated in this work (see legend) as a function of the applied external EF strength. Vertical dotted lines mark the separation between the regions where a “negative” (left) and a “positive” (right) EF is applied.

The two analyzed systems show markedly different responses. In the hydrogen-cyanide-containing system, an increase in the band gap is observed for fields that are concordant

to the natural dipole moment of the dimer system, while in the trimethylamine-bearing X-bonded complex there is a substantial *plateau* under that circumstance. When, on the other hand, the field is oriented against the natural dipole moment, both systems show a slight decrease of the band gap, a circumstance that again highlights the destabilization induced by intense EFs applied in opposition to the total dipole moment vector.

From a more qualitative point of view, the response of these systems can be visualized via the analysis of the HOMO and LUMO orbitals, displayed in Figs. S9 and S10 for the two X-bonded systems investigated in the current work. Although there are no profound differences in the case of the system composed of trimethylamine, the LUMO of the system containing hydrogen cyanide and experiencing a "negative" EF (Fig. S10, top panel) is deeply different from all others: as also visible from Fig. S8, indeed, the band gap of that system at -0.50 V/Å decreases with respect to its zero-field value, indicative of a lower stability, as also discussed in the previous section when the relative energies of the two systems were treated as a function of the intensity and direction of the applied EF.

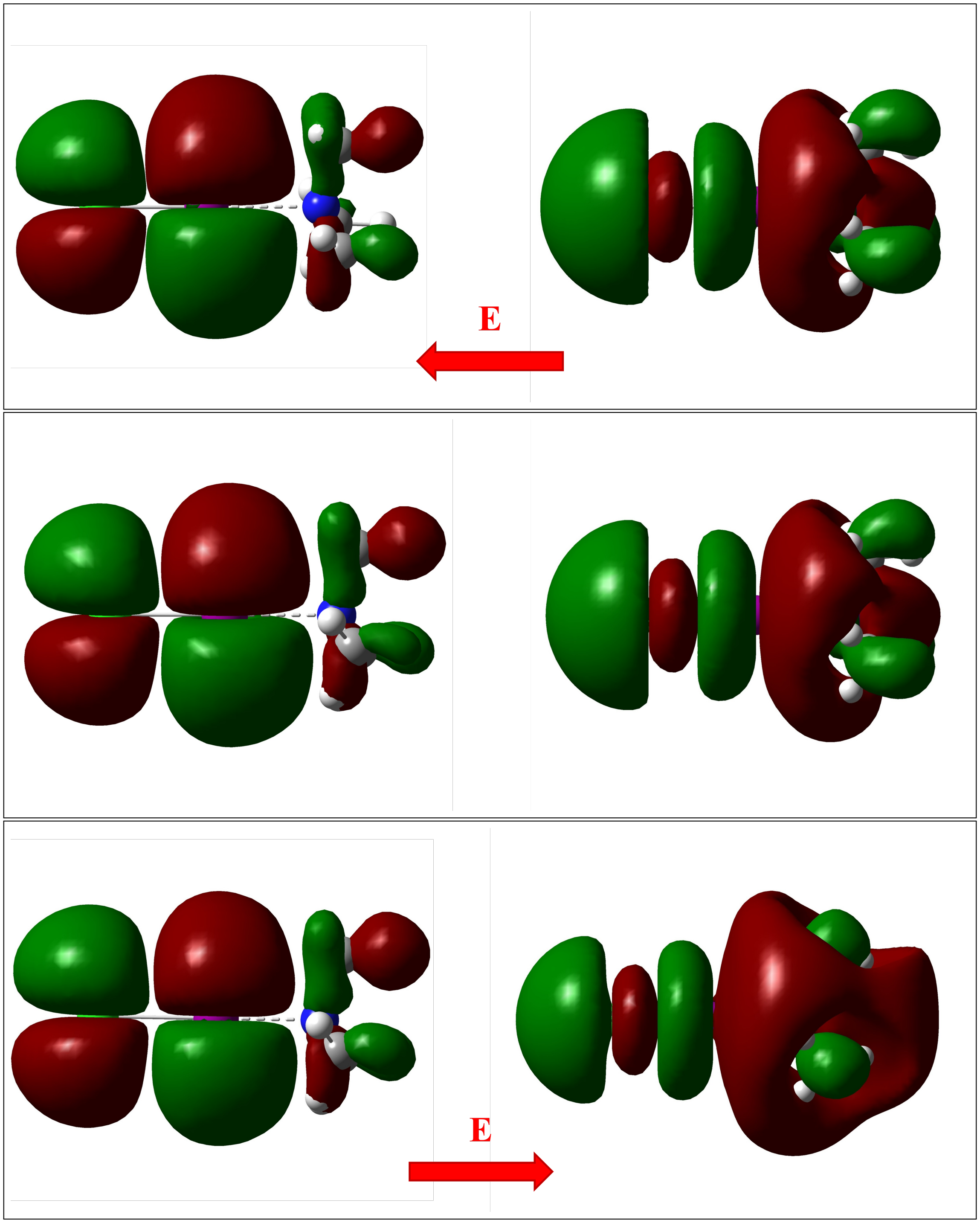

Figure S9: HOMO, left, and LUMO, right, of the system composed of ICl and trimethylamine. From top to bottom: -0.5 V/Å, 0 V/Å, +0.5 V/Å.

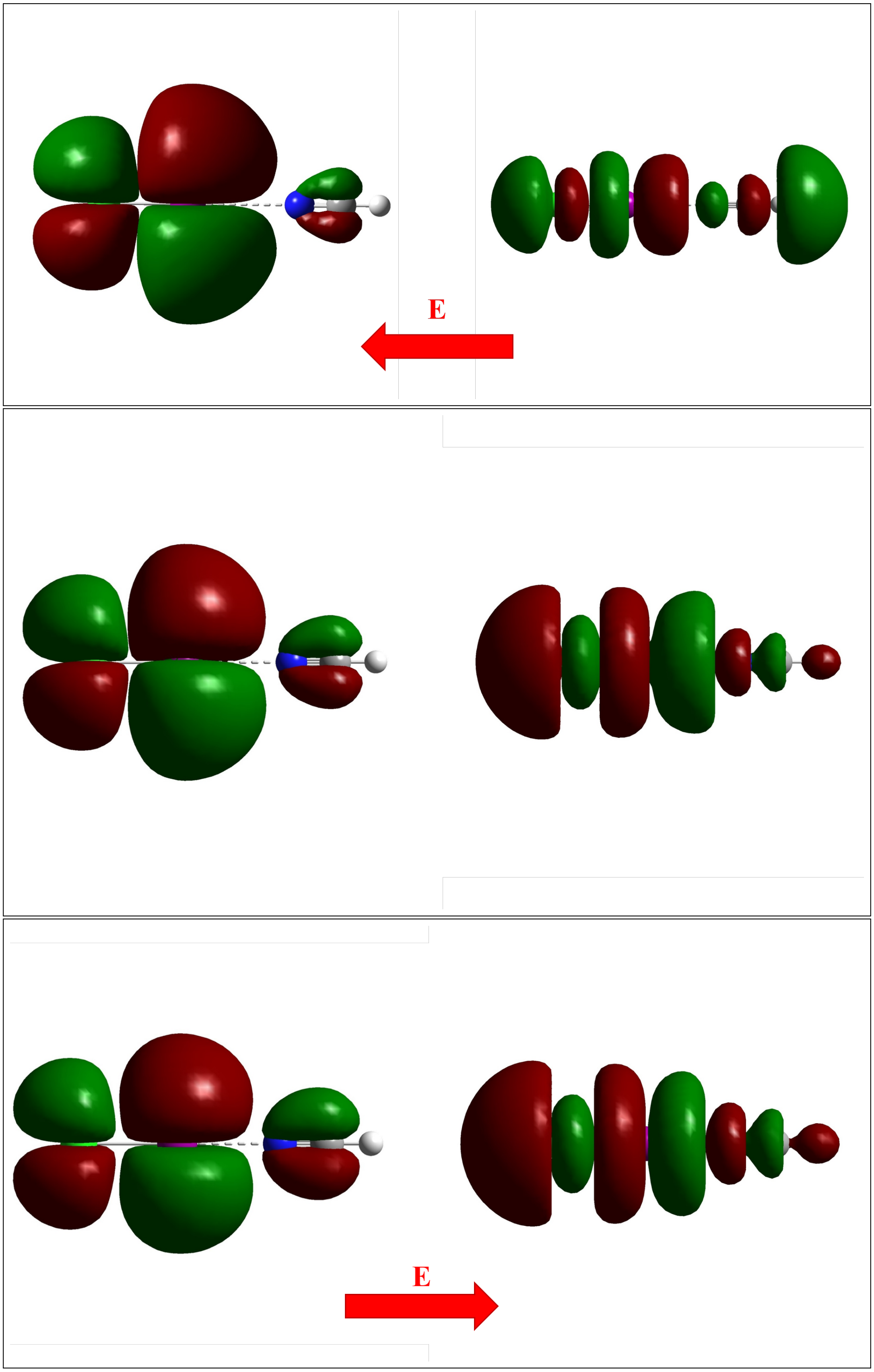

Figure S10: HOMO, left, and LUMO, right, of the system composed of ICl and hydrogen cyanide. From top to bottom: -0.5 V/Å, 0 V/Å, +0.5 V/Å.

References

(1) Amadeo, A.; Torre, M. F.; Mráziková, K.; Saija, F.; Trusso, S.; Xie, J.; Tommasini, M.; Cassone, G. Hydrogen Bonds under Electric Fields with Quantum Accuracy. *The Journal of Physical Chemistry A* **2025**, *129*, 4077–4092, PMID: 40298002.

(2) Nelson, R. D.; Lide, D. R.; Maryott, A. A. Selected values of electric dipole moments for molecules in the gas phase:. 1967.
